# Ferroptosis, necroptosis, and pyroptosis in anticancer immunity

**DOI:** 10.1186/s13045-020-00946-7

**Published:** 2020-08-10

**Authors:** Rong Tang, Jin Xu, Bo Zhang, Jiang Liu, Chen Liang, Jie Hua, Qingcai Meng, Xianjun Yu, Si Shi

**Affiliations:** 1 Department of Pancreatic Surgery, Fudan University Shanghai Cancer Center, No. 270 Dong’An Road, Shanghai, China; 2 Department of Oncology, Shanghai Medical College, Fudan University, Shanghai, China; 3 Shanghai Pancreatic Cancer Institute, No. 270 Dong’An Road, Shanghai, 200032 China; 4 Pancreatic Cancer Institute, Fudan University, Shanghai, China

**Keywords:** Necroptosis, Ferroptosis, Pyroptosis, Anticancer immunity

## Abstract

In recent years, cancer immunotherapy based on immune checkpoint inhibitors (ICIs) has achieved considerable success in the clinic. However, ICIs are significantly limited by the fact that only one third of patients with most types of cancer respond to these agents. The induction of cell death mechanisms other than apoptosis has gradually emerged as a new cancer treatment strategy because most tumors harbor innate resistance to apoptosis. However, to date, the possibility of combining these two modalities has not been discussed systematically. Recently, a few studies revealed crosstalk between distinct cell death mechanisms and antitumor immunity. The induction of pyroptosis, ferroptosis, and necroptosis combined with ICIs showed synergistically enhanced antitumor activity, even in ICI-resistant tumors. Immunotherapy-activated CD8+ T cells are traditionally believed to induce tumor cell death via the following two main pathways: (i) perforin-granzyme and (ii) Fas-FasL. However, recent studies identified a new mechanism by which CD8+ T cells suppress tumor growth by inducing ferroptosis and pyroptosis, which provoked a review of the relationship between tumor cell death mechanisms and immune system activation. Hence, in this review, we summarize knowledge of the reciprocal interaction between antitumor immunity and distinct cell death mechanisms, particularly necroptosis, ferroptosis, and pyroptosis, which are the three potentially novel mechanisms of immunogenic cell death. Because most evidence is derived from studies using animal and cell models, we also reviewed related bioinformatics data available for human tissues in public databases, which partially confirmed the presence of interactions between tumor cell death and the activation of antitumor immunity.

## Introduction

The mechanism by which the host immune system recognizes and kills tumor cells has not been well established. Whether dead tumor cells have immunogenic potential to elicit effective antitumor responses remains controversial because only nonself antigens are able to induce an immune response according to the “self/nonself” model that emerged in the 19th century [1]. Between the 1960s and 1980s, many studies reported that some treatment modalities, such as chemotherapy and radiotherapy, endow cancer cells with the ability to promote potent anticancer immunity [2, 3]. However, these findings were not widely acknowledged due to the lack of direct molecular evidence of tumor-associated antigen (TAA) involvement in antitumor immunity. In the 1990s and 2000s, several studies gradually elaborated TAA-directed antitumor immunity, ranging from how TAA-specific T cells survive negative selection in the thymus to how these T cells in the tumor microenvironment kill cancer cells [4, 5].

**Fig. 1:**
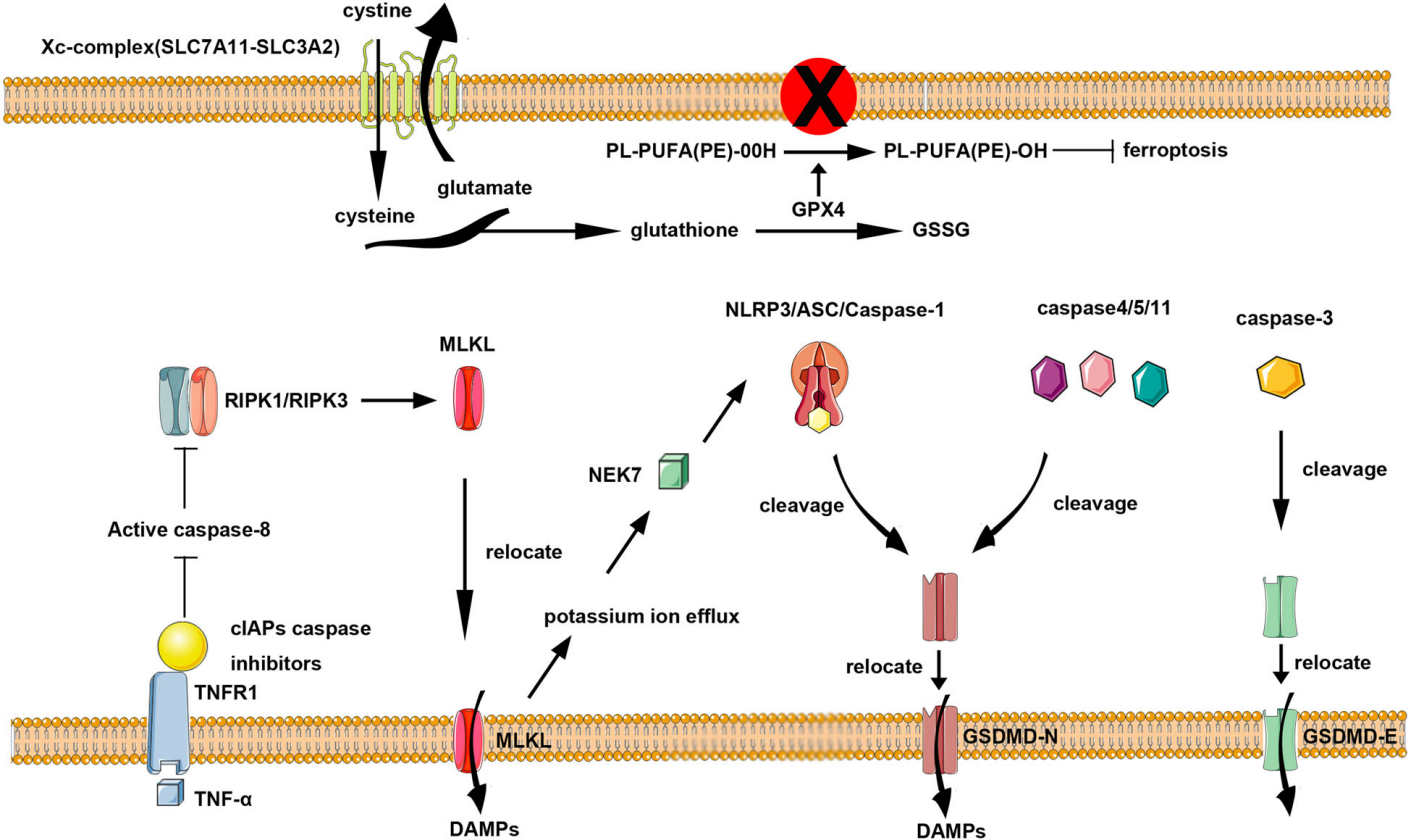
Pathways controlling ferroptosis, necroptosis, and pyroptosis. Xc-complex imports cystine, which is used to synthesize glutathione. Glutathione is used by GPX4 to prevent lipid reactive oxygen species accumulation. In this context, the normal expression and function of Xc-complex and GPX4 are essential for the inhibition of ferroptosis under physiological conditions. Gasdermins form membrane pores to cause pyroptosis. The following three pathways have been confirmed to induce pyroptosis in mammals: (1) NLRP3/ASC/caspase-1/GSDMD axis, (2) caspase-4/5/11/GSDMD axis, and (3) caspase-3/GSDME axis. Membrane-associated MLKL induces necroptosis. When the function of caspase-8 is inhibited, the binding of TNF-α and its receptor could promote the assembly of a RIPK1-RIPK3-MLKL signaling complex. RIPK3-mediated phosphorylation of MLKL leads to MLKL translocation to the plasma membrane to trigger membrane damage. As a result of membrane damage, potassium ion outflow could further activate NLRP3 through NEK7, which may be a crosstalk with pyroptosis pathway

The “danger model” of immunity, which emerged in the 1990s, partially attributes the activation of the antitumor immune response to nonphysiological cell death and the subsequent release of specific molecules, which are referred to as damage-associated molecular patterns (DAMPs). These DAMPs bind receptors in various immune cells and trigger a series of immune responses, including the activation of innate and adaptive immune cells [6], opsonization or phagocytosis of dying cancer cells [7], and maturation of dendritic cells (DCs) [8]. Over a long period, the cell death mechanisms have been inaccurately classified in a dichotomized manner as follows: (i) apoptosis regulated by intrinsic pathways and extrinsic intervention and (ii) accidental necrosis. However, apoptosis is usually regarded as an immune-tolerogenic process. In 2014, two studies reported a potential mechanism by which apoptotic cells maintain immune silence [9, 10]; the authors postulated that apoptotic caspases play a key role in the immune-tolerogenic process despite the lack of a clear molecular mechanism. Five years later, Jiang et al. elucidated that caspase 3/6/7 activation causes the downregulation of cGAS, MAVS, and IRF3, which are essential proteins for the activation of innate immunity [11]. Similarly, necrosis-induced inflammation facilitates only tissue repair responses (which are largely immunoregulatory) but not effective anticancer immunity [1]. In this context, researchers introduced a novel concept, i.e., immunogenic cell death (ICD), which might be elicited by tumor vaccination, radiotherapy, and some types of chemotherapy [12]. For a long time, ICD was also referred to as immunogenic apoptosis (IA) because most types of ICD occur via apoptosis. In recent years, with the increasing awareness of cell death mechanisms, many nonapoptotic cell deaths have been defined. Necroptosis, pyroptosis, and ferroptosis are three widely studied nonapoptotic cell deaths, all of which harbor unique molecular characteristics. Functionally, their role under physiological conditions in mammals has not been well-defined. According to previous studies, mammalian necroptosis and pyroptosis primarily exist to counteract pathogen infections and trigger an inflammatory anti-microbial response through the release of DAMPs. Interestingly, several lines of evidence suggest that crosstalk exists between necroptosis and pyroptosis [13–15]. Activated necroptosis by the induction of receptor-interacting protein kinase 1 (RIPK3) facilitates NLRP3-caspase-1-mediated IL-1β secretion [13]. Subsequent experiments supported that necroptosis signaling could trigger the RIPK3-mixed lineage kinase-like (MLKL)-NLRP3-Caspase-1 axis using MLKL and inflammasome knockout models [14]. In contrast to the deletion of core apoptotic effectors, the genetic deletion of the key necroptotic machinery, i.e., RIPK3 and MLKL, has no important influence on animal development. Ferroptosis can be induced by physiological conditions, such as high extracellular glutamate. Evolutionally, the incorporation of polyunsaturated fatty acids (PUFAs) into cell membranes is significant for the development of complicated neuronal circuits, modulating membrane fluidity, and cell adaptation to environments with different temperatures [16]. The accumulation of PUFAs in membranes creates a vulnerability to lethal lipid peroxidation due to their ability to form stabilized radicals, and many reactive electrophiles targeting nucleophilic sites in vital proteins are generated in this process. Hence, one possible physiological function of ferroptosis is the elimination of cells with the excessive production of electrophilic intermediates [17]. Another thought-provoking question relates to the identification of the factors that switch the cell death pattern from apoptosis to nonapoptotic cell death. Existing evidence suggests that necroptosis is a backup cell death mechanism triggered when apoptosis is hindered, which is highlighted by caspase-8 inhibition of lethal necroptotic signaling [18]. Acyl-CoA synthetase long-chain family member 4 (ACSL4) is a key factor that controls the sensitivity of cells to ferroptosis induction by regulating pro-ferroptotic lipid. Researchers unveiled ACSL4 as a target of caspase cleavage during bortezomib-induced apoptosis [19]. Therefore, it is biologically plausible that the inactivation of ACSL4 during apoptosis may inhibit the insertion of PUFAs into the membrane, thereby limiting the capability of cells to undergo ferroptosis. In contrast, cells that undergo ferroptosis because of cysteine deprivation have approximately 10% the normal level of intracellular GSH [20]. The reducing power of GSH may be required for the processing and activation of caspases 3 and 8 [21, 22]; hence, cells depleted of GSH could be unable to activate caspases. The main signal pathways of these nonapoptotic cell deaths are shown as Fig. 1. Many studies found that these cell death modalities were widely involved in cancer clearance. For example, genetically enhanced tumor-selective ferroptosis sensitivity obviously inhibited the formation and development of pancreatic cancer in genetically engineered mice [23]. This phenomenon was replicated through the administration of cyst(e)inase, which is a drug that depletes cysteine and cystine, suggesting a translatable means to inducing ferroptosis in pancreatic cancer [23]. Similarly, inducing necroptosis could dramatically increase the survival times of mice with orthotopic pancreatic cancer and reduce tumor growth, stroma, and metastasis [24]. In addition, pyroptosis induction eradicates neoplastic cells in multiple cancers [25]. Although these novel cell death modalities show obvious anticancer function based on many laboratory lines of evidence, whether they could affect the response of the immune system to tumors remains unclear. Some studies conducted over the past 5 years revealed that necroptosis, ferroptosis, and pyroptosis are tightly associated with antitumor immunity. Tumor cells undergoing necroptosis, ferroptosis, and pyroptosis could trigger robust anti-tumor immunity in vivo and in vitro, and their efficacy can be synergistically improved by immune checkpoint inhibitors (ICIs), even in ICI-resistant tumors. Coincidentally, the clinical use of ICIs has achieved great success in recent years [26, 27]. Regarding the mechanism, ICIs restrain tumor development by relieving the dysfunction of effector T cells. Immunotherapy-activated CD8+ T cells are traditionally believed to induce tumor cell death via the following two main approaches: (i) perforin-granzyme and (ii) Fas-FasL [28, 29]. However, many studies identified a new mechanism by which CD8+ T cells suppress tumors through the induction of ferroptosis and pyroptosis [30–33].

In this context, we compiled this review to summarize knowledge regarding the crosstalk among ferroptosis, pyroptosis, necroptosis, and antitumor immunity and highlight promising modalities for cancer treatment based on these novel findings.

## Traditional view of immunogenic cell death

The ability of dying cells to drive adaptive immunity depends on the following two major parameters: antigenicity and adjuvanticity [34, 35]. Antigenicity confers antigens with the ability to be recognized by host naive T cells. However, most naive T cells harboring antigens with high antigenicity are cleared by negative selection in the thymus during childhood [35]. The exception is some naive T cells expressing self-reactive low-affinity T cell receptors (TCRs) that evade thymic selection, implying that these antigens may initiate ICD once peripheral tolerance is disrupted. This finding partially explains why TAAs trigger an adaptive immune response under specific conditions [4, 36]. In addition, many non-self neoantigens are derived as TAAs from proteins harboring point mutations in tumors, which also confers tumor cells with high antigenicity [37]. Adjuvanticity is acquired from the spatiotemporally coordinated release of or exposure to DAMPs, which are necessary for the recruitment and maturation of antigen-presenting cells (APCs). Most therapy-induced antitumor immune responses observed in laboratories have been attributed to ICD [12, 38]. Nonetheless, many years elapsed before researchers established a widely acknowledged protocol for determining whether a specific type of cell death is categorized as ICD. The gold standard approach used to evaluate the capability of dying cells to induce adaptive immunity involves vaccination assays using immunocompetent, syngeneic mice [39]. Specifically, mouse-derived tumor cells are exposed to a potential ICD inducer ex vivo and then administered as a vaccine in the absence of any immunological adjuvant after the removal of exogenous chemical entities, such as the ICD inducer. After 7 to 10 days, mice are simultaneously treated with the vaccine and challenged with live cancer cells (at a minimal dose that is 100% effective in generating progressive lesions in naive mice). After 40 to 60 days, the tumor incidence and growth parameters are measured and compared with the control [39].

ICD features the spatiotemporally defined release of DAMPs (such as ATP and HMGB1), a type I IFN response, and the production of pathogen response-like chemokines that together enhance the immunogenic potential of dying cancer cells [40]. Different DAMPs mediate distinct immunostimulatory responses [41, 42]. For instance, surface-exposed HSPs not only trigger phagocytosis but also facilitate the formation of immunostimulatory Th1 and Th17 cells by inducing the secretion of proinflammatory cytokines from DCs [41]. In other cases, ATP release activates the NLRP3 inflammasome, resulting in the secretion of active IL1β, which is essential for ICD [42]. However, tumor cells with innate or experimentally enforced defects in pathways necessary for cell death-associated DAMP release, such as autophagy and the unfolded protein response, fail to undergo ICD in response to stimuli that would otherwise induce this process [35]. Therefore, extrinsic interventions applied to induce ICD have been widely researched in recent years. A qualified ICD inducer should be capable of promoting reactive oxygen species (ROS)-based ER stress in tumor cells [43, 44]. To date, many drugs approved for anticancer therapy, including doxorubicin, mitoxantrone, oxaliplatin, and bortezomib, have proven to be effective in inducing ICD as evidenced by vaccination experiments in mice [6, 45]. Other cancer therapy modalities, such as fractionated radiotherapy, but not single-dose therapy, exert optimal immunostimulatory effects, at least in mouse models [46].

However, over time, ICD was also termed IA because most ICD occurs via apoptosis. In recent years, many other cell death mechanisms distinct from apoptosis were reported to be associated with antitumor immunity, which is introduced in the following sections.

## Necroptosis and antitumor immunity

Necroptosis is a form of programmed cell death that occurs downstream of PRK1 and RIPK3, which assemble into an oligomeric complex termed the necrosome [47]. Necroptotic cells undergo rapid membrane permeabilization through the executioner protein mixed-lineage kinase-like (MLKL), thereby mediating the release of intracellular contents, including immunogenic DAMPs. Although accidental necrosis is widely regarded as a self-sacrifice strategy used by tumors to develop a favorable environment for proliferation and metastasis [48], its genetically programmed counterpart, i.e., necroptosis, has been reported to play a tumor inhibitory role in most cases [14, 49]. The levels of the RIPK3 protein were decreased in two thirds of a cohort of more than 60 cancer cell lines, suggesting that cancer cells prefer to evade necroptosis for survival. In addition, low RIPK3 expression indicates a worse prognosis in patients with multiple cancers [50, 51]. Drug-induced necroptosis directly inhibits tumor proliferation and reduces the incidence of metastasis through the accumulation of high ROS levels [14, 49, 52], which might be a rational explanation for the observed association between the expression level of necroptotic markers and patient survival.

Although tumor cell necroptosis appears to be a favorable factor for tumor clearance, some cells undergoing necroptosis are unable to explain the entire antitumor effect of necroptosis inducers, suggesting a potential connection between necroptosis and antitumor immunity [37, 53]. While cells undergoing necroptosis are involved in the activation of the immune system, particularly antigen presentation and cross-priming of CD8+ T cells [54, 55], in 2016, Aaes et al. were the first to confirm that necroptosis in tumors is ICD [56]. Necroptotic tumor cells were phagocytized, and then, bone marrow-derived DC maturation was induced [56]. Through in vivo and in vitro experiments, necroptotic tumor cells were shown to induce antitumor immunogenicity through the cross-priming and proliferation of CD8+ T cells [56]. Furthermore, the authors showed that necroptotic tumor cells serve as potent immunizers in a prophylactic tumor vaccination model, which is an essential step for confirming that the cell death type is immunogenic [35]. However, these researchers did not clarify the mechanism by which effector immune cells interact with necroptotic cells [56]. In a recent study, tumor control by necroptotic cells was shown to require BATF3+ cDC1 cells and CD8+ leukocytes [57]. In mice deficient in these two types of immune cells, necroptotic cells failed to trigger obvious anticancer effects. In contrast to the previous study by Aaes et al. [56], the authors of this study showed that immune-mediated tumor control by necroptotic fibroblasts requires nuclear factor κB (NF-κB) activation within dying cells but not MLKL-mediated and cell lysis-dependent DAMP release. In addition, in contrast to the traditional view of ICD [38], this study denied the contribution of newly primed CD8+ T cells from the tumor-draining lymph to tumor clearance and further implicated the local effects of necroptotic cells within the tumor microenvironment [57]. Notably, these authors observed a potential synergistic effect between necroptosis induction in the tumor microenvironment and immune checkpoint blockade (ICB) on promoting durable tumor rejection [57]. Interestingly, with advances in nanomedicine, a necroptotic cancer cell-mimicry nanovaccine was recently reported to boost antitumor immunity with a tailored immunostimulatory modality [58]. Vaccine-administered mice showed efficient lymph node trafficking and multiepitope T cell responses. This nanovaccine induced the expansion of IFN-γ-expressing CD8+ T cells and NKG2D+ natural killer (NK) cells. Importantly, the vaccination-induced tumor regression in vivo was optimized in combination with ICIs [58].

**Fig. 2:**
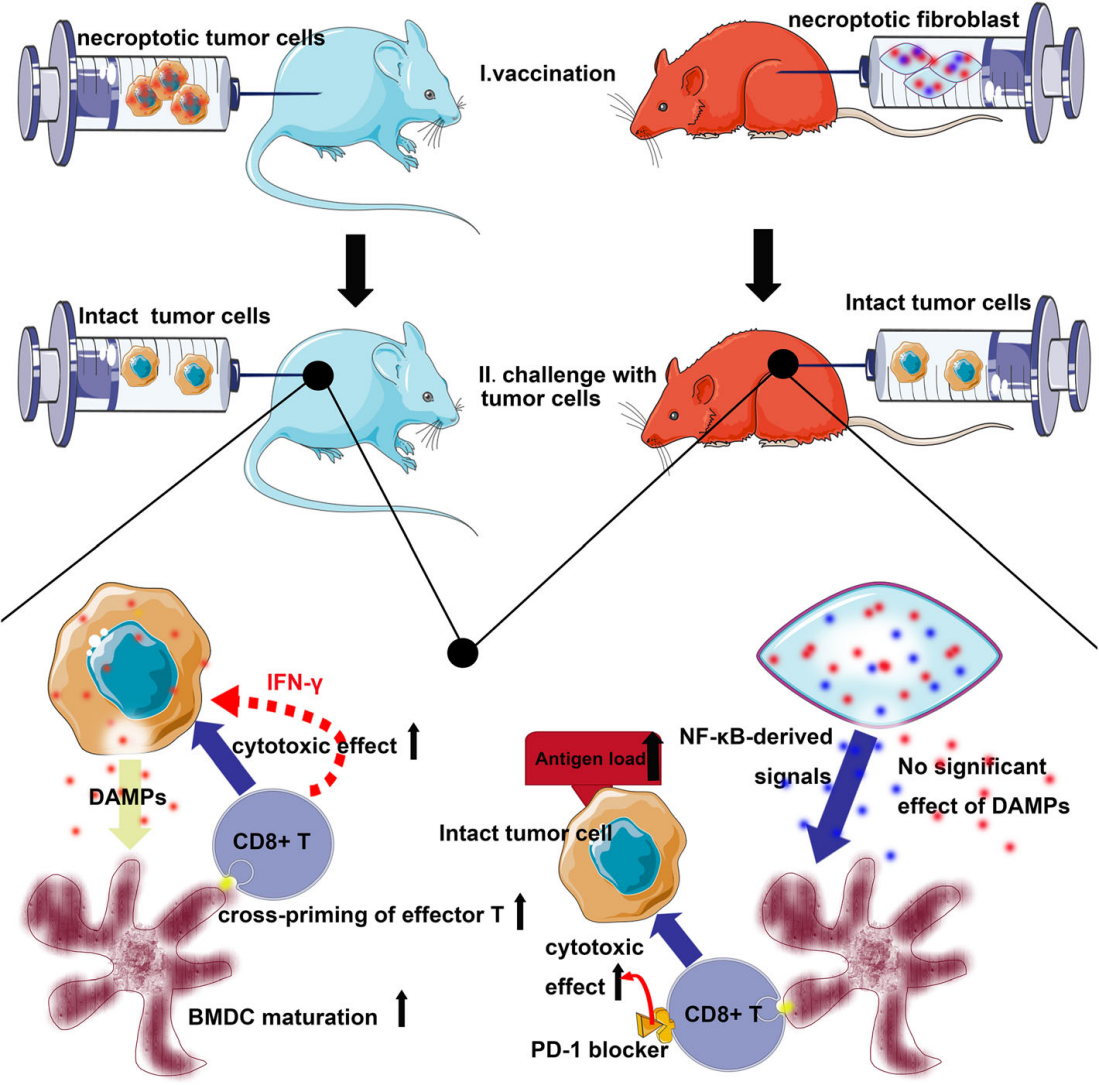
Crosstalk between necroptosis and antitumor immunity. Two strategies have been reported to trigger antitumor immunity through necroptosis. (1) Vaccination with necroptotic tumor cells: DAMPs released from tumor cells undergoing necroptosis promote the maturation of BMDCs, cross-priming of effector T cells, and subsequent cytotoxic effects. Excessive IFN-γ production is observed during this process, likely representing another anticancer approach used by CD8+ T cells. (2) Vaccination with fibroblasts: necroptotic cells release NF-κB-derived signals, further leading to DC activation, increased antigen loading, and robust CD8+ T cell-mediated tumor control. In this context, DAMPs do not appear to be involved in the activation of antitumor immunity. Tumor clearance is increased by the concomitant administration of PD1 inhibitors

The role of NF-κB activation in necroptosis-provoked antitumor immunity is controversial. As shown in the study by Yatim et al., robust cross-priming requires RIPK1 signaling and NF-κB-induced transcription within dying cells [55], and decoupling NF-κB signaling from necroptosis reduces the priming efficiency and the antitumor immune response. Similarly, Snyder and colleagues identified the important role of NF-κB-derived signals in DC activation and subsequent anticancer immunity [57]. However, Aaes et al. failed to correlate the immunogenicity of necroptotic tumor cells with the NF-κB activation status in vitro [56]. A potential explanation for the observed discrepancy may lie in the differences between the dying cells administered as a vaccine (PBS-washed dead tumor cells in the study by Aaes et al. and live fibroblasts in the studies by Yatim et al. and Snyder et al.). Interestingly, in mice with tumor-infiltrating immune cells lacking the expression of pattern recognition receptors capable of recognizing DAMPs, the administration of necroptotic fibroblasts into tumor tissues still controls tumor outgrowth, which reduces the importance of DAMPs in necroptosis-activated tumor immunity [57]. The crosstalk between necroptosis and anticancer immunity is summarized in Fig. 2.

## Ferroptosis and antitumor immunity

Ferroptosis is a novel form of regulated cell death characterized by the iron-dependent accumulation of lipid ROS to lethal levels [59]. The sensitivity to ferroptosis is determined by many essential molecules. Acyl-CoA synthetase long-chain family member 4 (ACSL4) dictates ferroptosis sensitivity by shaping the cellular lipid composition [60]. Mechanistically, ACSL4 enriches cellular membranes with long polyunsaturated ω6 fatty acids, which are vulnerable to ferroptosis execution [60]. Glutathione peroxidase 4 (GPX4), a selenoprotein harboring a selenocysteine (Sec) in its catalytic center, is required for an efficient reduction in peroxidized phospholipids [61, 62]. Hence, a low GPX4 expression level is associated with an increased sensitivity to ferroptosis [63]. Similarly, the activity of a cystine-glutamate antiporter (system Xc-) is important for ferroptosis execution. Small compounds, such as erastin, exclusively bind system Xc- and hinder the cellular influx of cystine, which further obstructs glutathione synthesis and increases the sensitivity to ferroptosis. Recent studies also defined ferroptosis as a type of autophagy-dependent cell death [64]. Hou et al. reported the contribution of autophagy to ferroptosis by degrading ferritin in fibroblasts and cancer cells [65]. The knockout or knockdown of the key gene controlling autophagy limits erastin-induced ferroptosis by decreasing the intracellular ferrous iron levels [65]. Moreover, lipophagy-mediated lipid droplet degradation promotes lipid peroxidation in ferroptosis, which is reversed by the knockdown of RAB7A (cargo receptor of lipid droplets) and autophagy-associated gene 5 (ATG5) [64].

Many studies have implicated ferroptosis in carcinogenesis [66–68]. Strategies manipulating the induction of ferroptosis effectively repress tumor development, even in some chemoresistant tumors [68, 69]. The tumor suppressor p53 is closely associated with the sensitivity to ferroptosis [70, 71]. In p53-intact mice, p53 binds the *SLC7A11* promotor region and inhibits its transcription [71], which is essential for ferroptosis induction. However, mice with multiple mutations in acetylation sites within p53 (K98R, K117R, K161R, and K162R) show a marked loss of p53-dependent ferroptotic responses [71]. Based on the widespread p53 mutations in distinct cancers [72], ferroptosis is speculated to be an intrinsic mechanism of resisting tumor initiation.

**Fig. 3:**
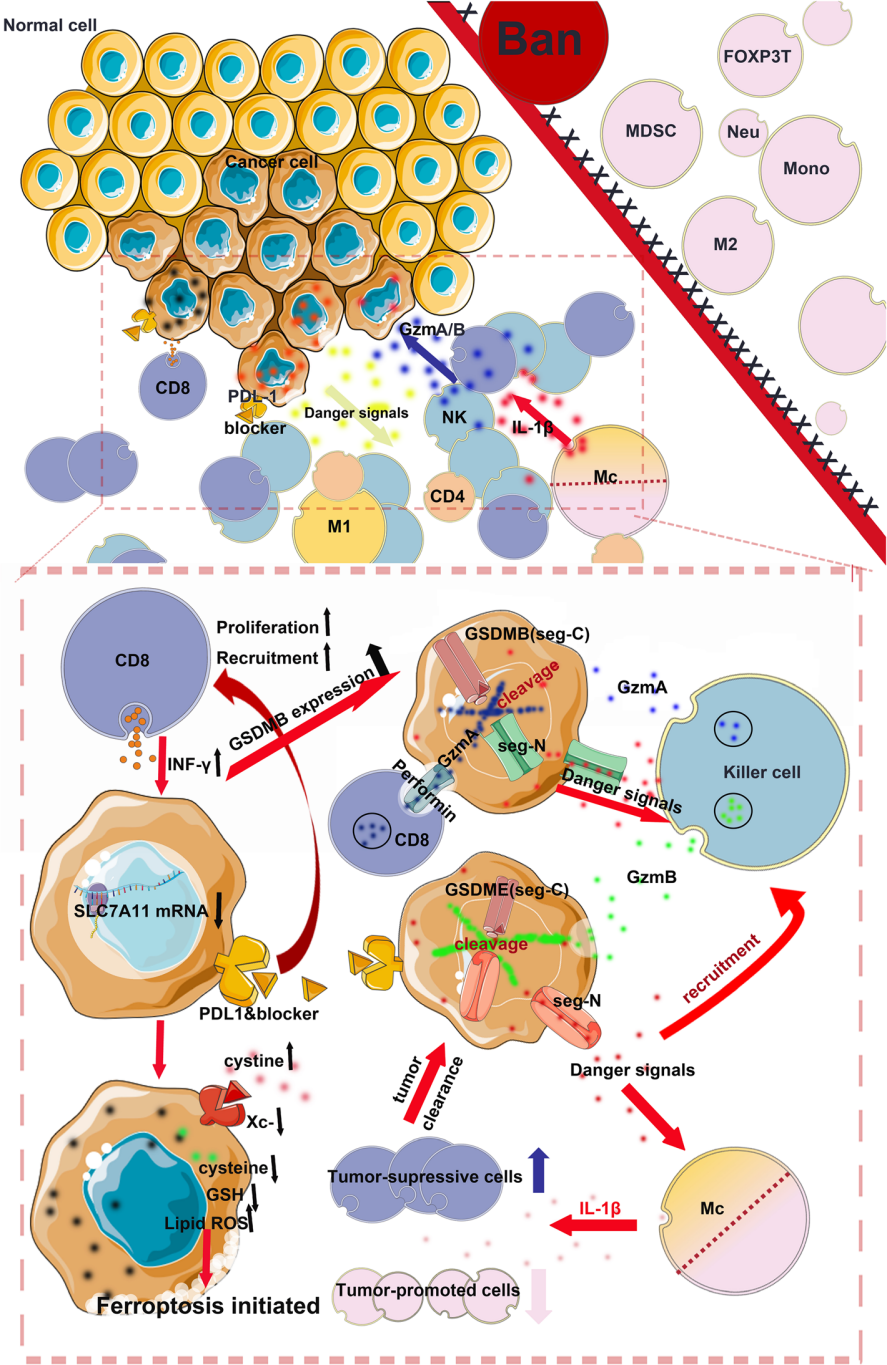
Crosstalk between ferroptosis and pyroptosis and antitumor immunity. Pyroptosis in less than 15% of tumor cells is sufficient to clear an entire tumor graft, suggesting that robust anticancer immunity plays an important role in pyroptosis-initiated tumor killing. On the one hand, tumor cells undergoing pyroptosis facilitate the recruitment of anticancer immune cells, including CD8+ T cells and NK cells, by releasing danger signals. However, the level of infiltration of tumor-promoting cells, such as MDSCs, is significantly decreased during this process. On the other hand, CD8+ T cells and NK cells induce cancer cell pyroptosis by secreting GzmA and GzmB, which are enzymes capable of cleaving GSDMB and GSDME, respectively. Activated macrophage-derived IL-1β is required for the antitumor immunity induced by tumor cell pyroptosis. Similarly, CD8+ T cells induce tumor cell ferroptosis by secreting IFN-γ, which mediates the downregulation of SLC7A11 and leads to the accumulation of lipid ROS. Notably, PD1/PDL-1 inhibitors exert an obvious synergistic effect with pyroptosis/ferroptosis inducers on tumor inhibition

Previous studies have investigated the role of ferroptosis in cancer under the following two themes: (i) the up/downregulation of specific signaling pathways that sensitize/desensitize tumor cells to ferroptosis induction [73, 74] and (ii) drugs or noncoding RNAs that induce ferroptosis in tumor models [75–77]. However, few studies reported the direct crosstalk between ferroptosis and antitumor immunity, although a biologically plausible hypothesis is that dying cells communicate with immune cells through a set of signals, such as the “find me” and “eat me” signals produced during cell death [78]. Cancer cells undergoing ferroptosis release HMGB1 in an autophagy-dependent manner [79, 80]. As a significant DAMP, HMGB1 is a key protein required for the immunogenicity of cancer cells [81]. Nevertheless, direct evidence of the connection between ferroptosis and antitumor immunity was not available until Wang et al. reported that CD8+ T cells induce ferroptosis in tumor cells in vivo [33]. Immunotherapy-activated CD8+ T cells downregulate the expression of SLC7A11, which is a molecule required for ferroptosis induction. CD8+ T cell-derived IFN-γ increases the binding of signal transducer and activator of transcription 1 (STAT1) to the SLC7A11 transcription start site, subsequently inhibiting its transcription. STAT1 deficiency in tumor cells abolishes the IFN-γ-mediated downregulation of SLC7A11 and reverses RSL3-induced lipid peroxidation and cell death [33]. In contrast, ferroptosis-resistant or ferroptosis inhibitor-treated tumor cells are insensitive to a PD-L1 inhibitor treatment. Further in vivo experiments revealed that T cells induce ferroptosis in mice bearing ovarian tumors [33]. Immunohistochemical studies have shown that the level of CD8 is negatively associated with Xc- complex expression, suggesting that the sensitivity to ferroptosis is parallel to anticancer immunity. Subsequently, the same team reported that IFN-γ derived from immunotherapy-activated CD8+ T cells synergizes with radiotherapy-activated ataxia-telangiectasia mutated (ATM) to induce ferroptosis in human fibrosarcoma cells and melanoma cells [32], which strengthened the status of ferroptosis among common anticancer modalities. However, these studies failed to elucidate the mechanism by which tumor cells undergoing ferroptosis enhance antitumor immunity. Because HMGB1 was recently reported to be a ferroptosis-related DAMP [79], the mechanism by which ferroptotic cells trigger potent immune responses may share some similarities with traditional ICD [82]. Unfortunately, due to the lack of evidence in the prophylactic tumor vaccination model, which is the gold standard for ICD detection, the definition of ferroptosis as an ICD is premature, despite its promising potential. While these findings indicate that ferroptosis has a synergistic effect on antitumor immunity, some theoretical discrepancies require additional investigation. Tumor cells undergoing ferroptosis might conceivably function as arachidonic acid (AA) donors for the transcellular biosynthesis of eicosanoids, thereby participating in the generation of biologically active immunomodulatory AA metabolites that affect antitumor immunity [83]. In addition, based on accumulating evidence, the increased intratumor production of prostaglandin E2 (PGE2) facilitates tumor evasion of immune surveillance [84, 85]. The induction of ferroptosis in tumor cells is associated with an increased expression of PTGS2 and the release of PGE2 [62]. Hence, PGE2 production may be an intrinsic obstacle to the induction of a robust immune response by ferroptotic cells. The crosstalk between ferroptosis and anticancer immunity is summarized in Fig. 3.

## Pyroptosis and antitumor immunity

As a lytic and inflammatory type of regulated cell death, pyroptosis is characterized by cell swelling, lysis, and the release of many proinflammatory factors, including IL-1β, IL-18, ATP, and HMGB1. Dying cells activate pyroptosis through the following two main approaches: (i) GSDMD (gasdermin D)-dependent activation regulated by caspase 1/4/5/11 and (ii) GSDME-dependent activation regulated by caspase 3 [86–90]. Activated caspases cleave the hinge region between the N- and C-terminal domains of GSDMD or GSDME, releasing the segment with lethal activity and leading to pyroptosis [91, 92]. Few studies investigated the roles of other GSDM family members in pyroptosis, but GSDMA, GSDMB, and GSDMC also harbor a pore-formation domain and can induce pyroptosis.

Because the activation of pyroptosis leads to the release of inflammatory mediators, such as IL-1 and IL-18, which might promote cancer development and progression [93–95], some researchers have viewed pyroptosis as another protumorigenic mechanism of cell death similar to accidental necrosis [93, 95]. Gao et al. reported significantly increased levels of the GSDMD protein in non-small cell lung cancer compared to matched adjacent specimens. Higher GSDMD expression has been associated with aggressive traits, including a larger tumor size and more advanced TNM stage in lung cancer [96]. Nonetheless, the exogenous activation of pyroptosis has recently been shown to elicit robust antitumor activity [97, 98]. Cancers of the digestive [99–102], respiratory [103, 104], reproductive [105, 106], and hemopoietic systems are sensitive to pyroptosis induction [107, 108]. Chemotherapeutic drugs, such as paclitaxel and cisplatin, effectively inhibit tumor proliferation and metastasis by inducing pyroptosis [109–111]. Interventions with certain chemotherapeutic drugs evoke a switch from caspase 3-dependent apoptosis to pyroptosis [111, 112]. As tumor cells show innate resistance to apoptosis, the development of new strategies aiming to induce pyroptosis may provide more efficient cancer therapy options and improve patient survival [113, 114]. Interestingly, although many studies have reported the role of pyroptosis in cancer, the association between pyroptosis and anticancer immunity remains unclear. Recently, two simultaneously published studies reported that tumor cells undergoing pyroptosis recruit tumor-suppressed immune cells [30, 31]. Wang et al. constructed a bioorthogonal system to reveal that pyroptosis in less than 15% of tumor cells was sufficient to clear an entire tumor graft operating in live animals. This bioorthogonal system enabled the controlled release of a drug from an antibody-drug conjugate in mice. When combined with nanoparticle-mediated delivery, desilylation catalyzed by Phe-BF3 could release a client protein, including an active gasdermin, from a nanoparticle conjugate selectively into tumor cells in mice. Another study conducted by Zhang et al. showed that in the pyroptosis-activated immune microenvironment, CD8+ T cells and NK cells reciprocally induce pyroptosis in tumor cells via granzyme B (an enzyme capable of cleaving GSDME), thereby establishing a positive feedback loop. However, the tumor suppression was abrogated in perforin-deficient mice or mice depleted of killer lymphocytes. These authors also showed that 20 of the 22 tested cancer-associated GSDME mutations reduce GSDME function, suggesting that GSDME inactivation is a strategy developed by cancer cells to escape immune attack.

Similarly, CD8+ T cells and NK cells were recently shown to trigger tumor clearance through the GSDMB-granzyme A axis, and this process is enhanced by IFN-γ [115]. Researchers confirmed that the expression of GSDMB, but not other GSDMs, induced pyroptosis through a mechanism facilitated by granzyme A. In fact, all findings combined indicate that the mechanism of pyroptosis induction by NK cells may vary among different cell lines, suggesting that tumor cells with different molecular characteristics potentially dictate the activation of the respective GSDM-granzyme axis [30, 31, 115]. Additional experimental evidence is needed to support this hypothesis. Indeed, an in vitro study previously implicated GSDMD as essential for the antitumor function of CD8+ T cells [103]. The colocalization of GSDMD and granzyme B was observed in the proximity of immune synapses, and a GSDMD deficiency reduced the cytolytic capacity of CD8+ T cells. Perforin was postulated to be the only pore-forming protein used by CD8+ T cells [116], but the authors proposed that GSDMD may be a new pore-forming protein that can be harnessed by effector T cells and form pores within mammalian cells, although the mechanism by which GSDMD is delivered into tumor cells from CD8+ T cells remains unclear.

In most cancer types, ICIs are significantly limited by the fact that approximately only one third of patients are responsive [117]. Tumors resistant to ICIs are deemed “cold” [118, 119]. However, Wang et al. found that ICIs efficiently killed cold tumor cells only in the context of the concomitant induction of pyroptosis. Similarly, pyroptosis induction alone failed to trigger efficient tumor inhibition, highlighting the importance of treating cold tumors with a combination of pyroptosis inducers and ICIs. Nonetheless, pyroptosis induction may not benefit all immunotherapy modalities. Chimeric antigen receptor (CAR) T cells were recently shown to rapidly activate extensive caspase 3/GSDME-dependent pyroptosis in targeted cells through the release of granzyme B. Consequently, pyroptosis-released factors activate caspase 1, which cleaves GSDMD in macrophages, leading to cytokine release and subsequent cytokine release syndrome (CRS), which is a severe adverse reaction characterized by fever, hypotension, and respiratory insufficiency [120]. The authors also found that the amount of perforin/granzyme B used by CAR T cells rather than existing CD8+ T cells is critical for the induction of target cell pyroptosis. Hence, the administration of a combination of ICIs and pyroptosis inducers for the treatment of solid tumors may not cause severe side effects because CAR T cells are not involved. The crosstalk between pyroptosis and anticancer immunity is summarized in Fig. 3.

## Approved drugs that induce necroptosis, ferroptosis, and pyroptosis

The development of new anticancer drugs targeting these novel mechanisms of cell death for clinical application is a lengthy process. Hence, studies exploring the effects of approved drugs with a known ability to induce ICD in patients with cancer are meaningful (Table 1). For example, artesunate, a widely prescribed antimalarial medicine, was reported to induce necroptosis and ferroptosis in tumor cells. Many clinical trials have reported the benefit of artesunate alone or in combination with other anticancer drugs in the treatment of cancer [75, 149]. Calreticulin exposure, autophagic ATP release, and HMGB1 upregulation are postulated to be the major mechanisms by which traditional chemotherapeutics trigger anticancer immunity. In addition, some chemotherapy drugs, including sorafenib, cisplatin, and paclitaxel, induce ferroptosis and pyroptosis, prompting a careful re-evaluation of the type of cell death induced by chemotherapy.

**Table 1:** Summary of clinically approved drugs that may induce ferroptosis, necroptosis, and pyroptosis in cancers and their effects on antitumor immunity

Drug name	Daily use	Target	Effect on tumor cell death	References	Effect on antitumor immunity	References
Sulfasalazine	Anti-inflammatory drug	System Xc-	Ferroptosis induction	[121]	Unknown	
Glutamate	Nutrient	System Xc-	Ferroptosis induction	[59]	Increased immune suppression	[122]
Sorafenib	Anticancer drug	System Xc-	Ferroptosis/necroptosis induction	[123]	Null effect on antitumor immunity	[124]
Cisplatin	Anticancer drug	GSH/GSDME	Ferroptosis/pyroptosis induction	[109, 125]	Enhanced antitumor immunity	[126]
Statins	Hyperlipemia drug	HMGCR	Ferroptosis induction	[127]	Enhanced antitumor immunity	[128]
Trigonelline	Nutrient additive	Nrf2	Ferroptosis induction	[129]	Unknown	
Artesunate	Antimalarial drug	ROS	Ferroptosis/necroptosis induction	[75]	Enhanced antitumor immunity	[130–132]
Shikonin	Thrombocytopenia drug	RIPK1/RIPK3	Necroptosis induction	[52]	Enhanced antitumor immunity	[133]
Resibufogenin	Heart failure drug	RIPK3, MLKL	Necroptosis induction	[134]	Unknown	
5-FU	Anticancer drug	TNF-α/RIPK3	Necroptosis induction	[135]	Enhanced antitumor immunity	[136]
Metformin	Anti-diabetes drug	GSDMD	Pyroptosis induction	[137]	Enhanced antitumor immunity	[138]
Anthocyanin	Nutrient	NLRP3	Pyroptosis induction	[139]	Enhanced antitumor immunity	[140]
DHA	Nutrient	GSDMD	Pyroptosis induction	[141]	Enhanced antitumor immunity	[142]
Paclitaxel	Anticancer drug	GSDME	Pyroptosis induction	[109]	Enhanced antitumor immunity	[143]
Iron	Nutrient	Ferrous/GSDME	Ferroptosis/necroptosis induction	[144, 145]	Enhanced antitumor immunity	[146]
Doxorubicin	Anticancer drug	GSH/GSDME2	Ferroptosis/pyroptosis induction	[147]	Enhanced antitumor immunity	[148]

Although the drugs listed in Table 1 have shown moderate efficacy in treating cancer, given their potential role in inducing ICD, we hypothesize that appropriate combinations of these drugs with ICIs may achieve greater therapeutic benefits. Many studies have reported the increased benefit of combinations of chemotherapy and ICIs in cancer therapy (Table 2) [124, 150–152]. Based on the results of the KEYNOTE-189 trial involving patients with metastatic non-squamous non-small cell lung cancer, the addition of an ICI to standard chemotherapy with pemetrexed and a platinum-based drug results in significantly longer overall and progression-free survival than chemotherapy alone [150]. Similarly, in another trial (IMpower150), improved survival was noted among patients treated with atezolizumab plus chemotherapy compared with patients treated with chemotherapy alone regardless of the presence of liver metastasis or epidermal growth factor receptor mutations [151].

**Table 2:** Summary of published clinical trials combining immune checkpoint inhibitors with chemotherapy/radiotherapy in cancer treatment

Treatment modality	Trial number	Cancer type	Potential nonapoptotic cell death pathway	Main conclusion
Pembrolizumab (anti-PD-L1) + pemetrexed and a platinum-based drug (chemotherapy)	NCT02578680	Non-small cell lung cancer	Ferroptosis/pyroptosis/necroptosis	In patients with previously untreated metastatic non-squamous NSCLC without EGFR or ALK mutations, the addition of pembrolizumab to standard chemotherapy of pemetrexed and a platinum-based drug resulted in significantly longer overall survival and progression-free survival than chemotherapy alone.
Atezolizumab (anti-PD-L1) + bevacizumab plus carboplatin plus paclitaxel (chemotherapy)	NCT02366143	Non-small cell lung cancer	Ferroptosis/pyroptosis/necroptosis	Improved survival was noted among patients treated with immunotherapy + chemotherapy compared with those only given chemotherapy.
Atezolizumab (anti-PD-L1) + platinum-based chemotherapy	NCT02367781	Non-squamous non-small cell lung cancer	Ferroptosis/pyroptosis/necroptosis	Improved survival was noted among patients treated with immunotherapy + chemotherapy compared with those only given chemotherapy.
Atezolizumab (anti-PD-L1) + platinum (chemotherapy)	NCT02807636	Locally advanced or metastatic urothelial carcinoma	Ferroptosis/pyroptosis/necroptosis	The use of atezolizumab plus platinum-based chemotherapy as a potential first-line treatment option for metastatic urothelial carcinoma.
Pembrolizumab (anti-PD-L1) + stereotactic body radiotherapy	NCT02492568	Advanced non-small cell lung cancer	Ferroptosis/necroptosis	The overall response rate is larger in the group with radiotherapy + immunotherapy; however, the positive results were largely influenced by the PD-L1-negative subgroup.
Pembrolizumab (anti-PD-L1) + docetaxel (chemotherapy)	NCT02574598	Non-small cell lung cancer	Necroptosis	The combination of pembrolizumab plus docetaxel was well tolerated and substantially improved the ORR and PFS in patients with advanced NSCLC who had previous progression after platinum-based chemotherapy.
Ipilimumab (anti-CTLA4) + stereotactic ablative radiation therapy	NCT02239900	Metastatic lesions in the liver or lung	Ferroptosis/necroptosis	This phase II trial of ipilimumab with stereotactic radiotherapy describes satisfactory outcomes and low toxicities, lending support to the further investigation of combined-modality therapy for metastatic cancers.

In addition to cytotoxic chemotherapy drugs, some non-toxic reagents may induce anticancer immunity. Cancer-derived oxysterols have consistently been shown to generate an immunosuppressive tumor microenvironment by dampening the DC antigen presentation ability, reducing the number of CD8+ T cells, and blocking the recruitment of LOX-1-positive MDSCs, which perform pro-tumor functions. Hence, reagents that block cholesterol synthesis may stimulate the host antitumor immune response and synergistically improve the efficacy of ICIs. Actually, many clinical observations have supported this hypothesis. Patients who have taken statins for more than 5 years exhibit a 47% decreased risk of colorectal cancer than non-statin users after adjusting for confounding factors [153]. Statins reduce patient mortality and prolong the relapse-free survival of patients with various cancers regardless of whether the statins were taken before or after the cancer diagnosis [154–156]. Moreover, inhibitors of the mevalonate pathway, including statins, are robust cancer vaccinations and synergize with ICIs in multiple mouse cancer models [157]. Interestingly, statins were recently shown to induce ferroptosis in cancer cells by reducing the production of CoQ10, which desensitizes tumor cells to ferroptosis [127]. Hence, given the crosstalk between ferroptosis in cancer cells and activated anticancer immunity, statins should be highlighted as an important reagent for cancer therapy and a candidate adjuvant to immunotherapy.

The abscopal effect is a phenomenon in which local radiotherapy is associated with the regression of metastatic cancer at a distance from the irradiated site [158, 159]. In 2012, Postow et al. reported a case of the abscopal effect in a patient with melanoma treated with ipilimumab and radiotherapy [160]. Recently, radiotherapy was reported to kill tumor cells by inducing ferroptosis and necroptosis [32, 161]. A plausible hypothesis is that dying cancer cells release “danger signals” to recruit anticancer immune cells and enhance antigen presentation by DCs. Reciprocally, ICI-activated anticancer immunity might restore the structure of vessels in the tumor microenvironment, which relieves the hypoxia state of tumor cells and increases the efficacy of radiotherapy [162]. Hence, the combination of radiotherapy and immunotherapy is a promising modality for cancer treatment and is already supported by a well-designed clinical trial [163].

In addition, many nanoparticles, such as zero-valent iron nanoparticles and arginine-rich manganese silicate nanobubbles, induce ferroptosis in cancer cells depending on the nanoparticle structure and chemical modifications [164–167]. Similarly, nanoparticles trigger necroptotic tumor cell death by modulating autophagy [168]. Although most nanoparticle-based treatments have not been approved for clinical application to date, nanoparticles alone and as drug transporters and biomimetic nanotechnology have a bright future in next-generation cancer therapy [169, 170].

## Summary of the bioinformatic evidence of the roles of necroptosis, ferroptosis, and pyroptosis in anticancer immunity

Although laboratory studies have revealed the crosstalk between distinct cell death mechanisms and anticancer immunity, a substantial lack of evidence from human samples has hindered a better understanding of the potential for clinical translation.

**Fig. 4:**
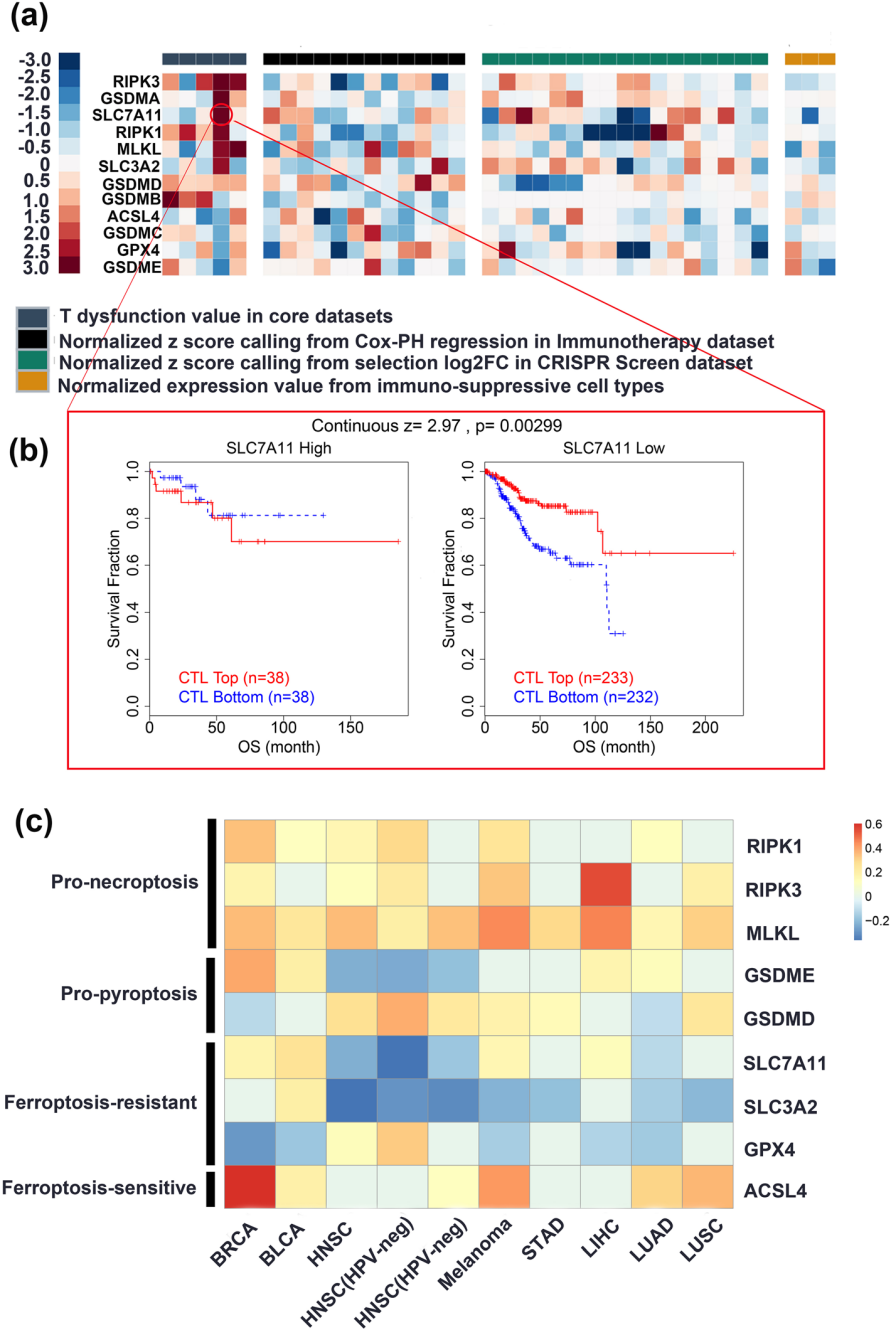
Bioinformatic evidence of the effects of necroptosis, ferroptosis, and pyroptosis on T cell dysfunction and CD8+ T cell infiltration based on molecular signatures. **a** The effects of necroptosis, ferroptosis, and pyroptosis on T cell dysfunction and CD8+ T cell infiltration were evaluated based on the molecular signatures of four sets with 37 independent cohorts (core cohorts, immunotherapy datasets, CRISPR screen datasets, and datasets of immunosuppressive cell types). The core cohorts consisted of the five most confident results obtained using gene expression data, and a high z-score (red) suggests that the indicated gene promotes T cell dysfunction. The immunotherapy datasets consisted of 12 datasets of patients who received either ICIs or ACT. In this set, a high *z*-score (red) represents an unfavorable role of the indicated gene in improving the effects of immunotherapy. CRISPR screening of mouse cancer cells identified genes whose knockout enhanced the efficacy of T cell-mediated tumor cell killing based on 17 cohorts; in these studies, a *z*-score < 0 (blue) reflects the downregulation of the indicated gene after an increase in either T cell function or the efficacy of immunotherapy, suggesting the negative effects of the indicated gene on immunotherapy outcomes. Immunosuppressive cells restrict the tumor infiltration of T cells, including cancer-associated fibroblasts (CAFs), myeloid-derived suppressor cells (MDSCs), and the M2 subtype of tumor-associated macrophages (TAMs). This section presents the gene expression levels in these T cell exclusion signatures, and a high *z*-score (red) indicates that the specified gene is overexpressed in immunosuppressive cells. **b** An example of the evaluation of T cell dysfunction. A considerable amount of CTL infiltration predicts prolonged survival only in tumor samples from patients with low SLC7A11 expression, suggesting that SLC7A11 potentially promotes T cell dysfunction. **c** The effects of necroptosis, ferroptosis, and pyroptosis on CD8+ T cell infiltration based on molecular signatures are shown

With the increasing number of RNA sequencing-based studies and advances in bioinformatic methods, we estimated the degree of infiltration of distinct immune cells and the enrichment of immunity-related signatures in the human tumor microenvironment. In this review, we systematically analyzed a panel of genes essential for necroptosis, ferroptosis, and pyroptosis to explore their roles in T cell dysfunction and CD8+ T cell infiltration. Specifically, we reviewed the published data in the Tumor Immune Dysfunction and Exclusion (TIDE) (http://tide.dfci.harvard.edu/query/) and Tumor Immune Estimation Resource (TIMER) (https://cistrome.shinyapps.io/timer/) databases [117, 171]. Overall, the relationship between key signatures of cell death and T cell dysfunction depends on the tumor type (Fig. 4a). For example, in an endometrial carcinoma cohort (TCGA-UCEC), a higher percentage of cytotoxic T lymphocytes (CTLs) predicted a longer overall survival only in patients with lower SLC7A11 expression, which parallels the results of increased anticancer immunity with increased ferroptosis sensitivity (Fig. 4b). Hence, the combination of ICIs and ferroptosis inducers may prolong the survival of patients with endometrial carcinoma. However, this relationship was absent in other core cohorts. Three molecules essential for the initiation of necroptosis, i.e., RIP1, RIP3, and MLKL, were associated with obvious T cell dysfunction in more than two core cohorts; however, their overexpression predicted a prolonged survival in many clinical studies of ICIs, suggesting that the detrimental role of necroptosis markers in T cell function is rescued by ICIs.

In addition, we calculated the correlation between CD8+ T cell infiltration and the expression of essential markers of distinct types of cell death (Fig. 4c). Then, we compared these correlations across seven common cancers and found that the pro-necroptosis, pro-pyroptosis, and pro-ferroptosis signatures were broadly associated with greater CD8+ T cell infiltration. In contrast, the anti-ferroptosis signatures were correlated with less CD8+ T cell infiltration. Because a greater enrichment of CD8+ T cells usually reflects better ICI efficacy [172], rational combinations of ICIs and agents that induce cell death may be effective anticancer modalities.

**Fig. 5:**
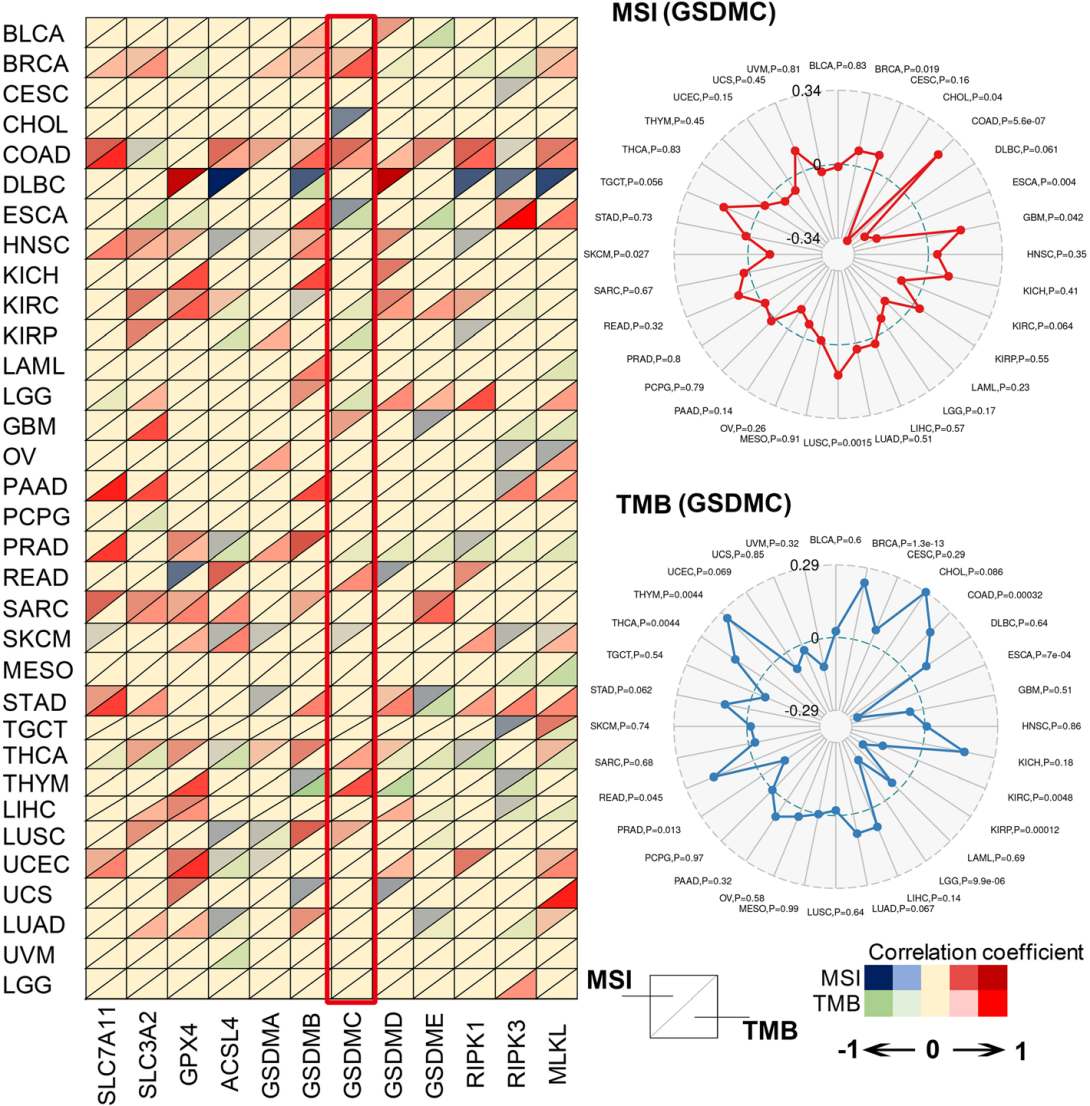
Correlations among ferroptosis-, pyroptosis-, and necroptosis-related genes; microsatellite instability (MSI); and the tumor mutation burden (TMB) across 33 cancers. The correlations among ferroptosis-, pyroptosis-, and necroptosis-related genes, MSI, and TMB were visualized as a heatmap. The colors of the up-pointing triangles reflect the correlation strength between the expression levels of ferroptosis-, pyroptosis-, and necroptosis-related genes and MSI. The colors of the down-pointing triangles reflect the correlation strength between the expression levels of ferroptosis-, pyroptosis-, and necroptosis-related genes and TMB. The correlations between GSDMC and MSI or TMB were further visualized as a radar map

Microsatellite instability (MSI) and the tumor mutation burden (TMB) are important predictive biomarkers for cancer immunotherapy. Therefore, we further explored the correlations among genes involved in necroptosis, ferroptosis, and pyroptosis, MSI, and TMB across 33 cancers, and the results are visualized in Fig. 5. The results revealed that the sensitivity of necroptosis, ferroptosis, and pyroptosis may be positively correlated with higher MSI and TMB only in a portion of cancers. For example, the expression of the GSDM family, which are the executors of pyroptosis, is positively associated with a higher MSI and/or TMB. In addition, increased MSI and TMB are correlated with RIPK1 and MLKL, which are key molecules in necroptotic cell death. Whether the sensitivity of ferroptosis is increased in tumor samples harboring higher MSI and TMB is unclear given that a ferroptosis-insensitivity marker (SLC7A11) is positively associated with MSI and TMB, while a ferroptosis-sensitivity marker (ACSL4) is also positively correlated with MSI and TMB. In contrast to the other members of the GSDM family, the role of GSDMC in anticancer immunity has not been reported to date; hence, we constructed a radar plot to highlight the relationship among GSDMC expression, MSI, and TMB. The results showed that the expression level of GSDMC is positively correlated with MSI or TMB in seven cancers (BRCA, COAD, READ, GBM, THCA, THYM, and LUSC) but negatively correlated with MSI or TMB in seven other cancers (CHOL, ESCA, KIRC, KIRP, LGG, PRAD, and SKCM). The activation of GSDMB to induce pyroptosis in some specific cancers may be a promising modality to improve the efficacy of ICIs given the positive correlation between GSDMB expression and MSI or TMB in these cancers. This hypothesis is expected to be validated by future studies.

## Conclusions

In patients with most cancer types, the application of ICIs is significantly limited by the fact that approximately only one third of patients are responsive. In this review, we reveal broad crosstalk between anticancer immunity and nonapoptotic cell death mechanisms based on existing laboratory and bioinformatic evidence. In this context, the concomitant induction of nonapoptotic tumor cell death is a promising strategy for treating cancer. A long-standing challenge is the design of potent drugs that specifically activate the above-mentioned nonapoptotic cell deaths in humans with rigorous safety testing. However, in the near future, medical organizations are encouraged to carry out clinical trials that treat patients with approved drugs that function in activating ferroptosis, pyroptosis, or necroptotic cell death with the concomitant use of ICIs. Although these drugs may not be pathway specific, the efficacy, safety, and side-effects of such combinations could provide valuable suggestions for future investigations.

## Data Availability

Not applicable.

## Abbreviations

AA: Arachidonic acid
ACSL4: Acyl-CoA synthetase long-chain family member 4
ATG5: Autophagy-associated gene 5
ATM: Ataxia-telangiectasia mutated
CAR: Chimeric antigen receptor
CRS: Cytokine release syndrome
CTL: Cytotoxic T lymphocytes
DAMP: Damage-associated molecular patterns
DC: Dendritic cell
GPX4: Glutathione peroxidase 4
GSDM: Gasdermin
HMGB1: High mobility group box 1
IA: Immunogenic apoptosis
ICIs: Immune checkpoint inhibitors
ICB: Immune checkpoint blockade
ICD: Immunogenic cell death
MLKL: Mixed-lineage kinase-like
MSI: Microsatellite instability
NF-κB: Nuclear factor κB
NK: Natural killer
PGE2: Prostaglandin E2
PRK: Receptor-interacting protein kinase
Sec: Selenocysteine
STAT1: Signal transducer and activator of transcription 1
System Xc-: Cystine-glutamate antiporter
TAA: Tumor-associated antigen
TCR: T cell receptors
TIDE: Tumor immune dysfunction and exclusion
TIMER: Tumor immune estimation resource
TMB: Tumor mutation burden
